# 53BP1 contributes to regulation of autophagic clearance of mitochondria

**DOI:** 10.1038/srep45290

**Published:** 2017-03-27

**Authors:** Cha Kyung Youn, Hong Beum Kim, Ting Ting Wu, Sanggon Park, Sung Il Cho, Jung-Hee Lee

**Affiliations:** 1 Laboratory of Genomic Instability and Cancer therapeutics, Cancer Mutation Research Center, Chosun University School of Medicine, 375 Seosuk-dong, Gwangju 61452, Republic of Korea; 2 Department of premedical Sciences, Chosun University School of Medicine, 375 Seosuk-dong, Gwangju 61452, Republic of Korea; 3 Department of Cellular and Molecular Medicine, Chosun University School of Medicine, 375 Seosuk-dong, Gwangju 61452, Republic of Korea; 4 Department of Internal Medicine, Hemato-oncology, Chosun University School of Medicine, 375 Seosuk-dong, Gwangju 61452, Republic of Korea; 5 Department of Otolaryngology-Head and Neck Surgery, Chosun University School of Medicine, 375 Seosuk-dong, Gwangju 61452, Republic of Korea

## Abstract

Autophagy, the primary recycling pathway within cells, plays a critical role in mitochondrial quality control under normal growth conditions and in the cellular response to stress. Here we provide evidence that 53BP1, a DNA damage response protein, is involved in regulating mitochondrial clearance from the cell via a type of autophagy termed mitophagy. We found that when either human or mouse cells were 53BP1-deficient, there was an increase in mitochondrial abnormalities, as observed through staining intensity, aggregation, and increased mass. Moreover, a 53BP1-depleted cell population included an increased number of cells with a high mitochondrial membrane potential (ΔΨm) relative to controls, suggesting that the loss of 53BP1 prevents initiation of mitophagy thereby leading to the accumulation of damaged mitochondria. Indeed, both 53BP1 and the mitophagy-associated protein LC3 translocated to mitochondria in response to damage induced by the mitochondrial uncoupler carbonyl cyanide m-chlorophenylhydrazone (CCCP). The recruitment of parkin, an E3-ubiquitin ligase, to mitochondria in response to CCCP treatment was significantly decreased in 53BP1-deficient cells. And lastly, using p53-deficient H1299 cells, we confirmed that the role of 53BP1 in mitophagy is independent of p53. These data support a model in which 53BP1 plays an important role in modulating mitochondrial homeostasis and in the clearance of damaged mitochondria.

Mitochondria are essential organelles involved in energy production, calcium homeostasis, Fe-S cluster biogenesis, metabolic biosynthetic pathways, and cell health and survival^1^,^2^,^3^,^4^. Mitochondrial dysfunction, or the accumulation of damaged mitochondria, can trigger programmed cell death, and has been implicated in the pathology of numerous human diseases, such as neurodegenerative diseases, diabetes, obesity, myopathies, cancer and aging^5^,^6^,^7^,^8^. Dysfunctional mitochondria are specifically targeted for autophagy and are engulfed by autophagosomes, which then fuse with lysosomes to form an autolysosome in which the mitochondria are degraded^9^,^10^. The process by which damaged mitochondria are removed through autophagy, also known as mitophagy, is a potential target for therapeutic interventions in mitochondrial disease pathophysiology.

Because genes located on both nuclear and mitochondrial DNA encode regulators of mitochondria, mutations in either DNA molecule may result in human disease^11^,^12^. Mitochondrial disorders are caused by spontaneous or inherited mutations, or by exogenous factors. However, whether the DNA damage directly promotes mitochondrial disorders or is simply a byproduct of the risk factors that promote mitochondrial disorders is unknown. Recent reports have linked the loss of DNA repair enzymes to metabolic defects, mitochondrial biogenesis, and mitochondrial DNA content^12^,^13^,^14^. During the process of ATP synthesis, mitochondria generates reactive oxygen species (ROS) from electron transport chain^15^. Owing to the close proximity of mitochondrial DNA to the electron transport chain, mitochondrial DNA is more oxidatively damaged than nuclear DNA^16^,^17^. Because mutations in mitochondrial DNA induce chronic mitochondrial stress, which alters the expression of genes related mitochondrial function and structure^18^,^19^,^20^, prevention and repair of mitochondrial DNA damage would be expected to have a central role in the mitochondrial homeostasis.

53BP1 is an important component of the DNA damage response (DDR) that that rapidly forms nuclear foci in response to DNA damage^21^. This process is dependent on the ATM/ATR-induced phosphorylation of histone H2AX (γ-H2AX)^22^,^23^. In addition to forming DNA damage-dependent foci, 53BP1 plays a pivotal role in defining the DNA double-strand break (DSB) repair pathway in the G1 and S/G2 phases of the cell cycle^24^,^25^,^26^,^27^. Interestingly, it was reported previously that 53BP1 localizes to both the cytoplasm and the nucleus^28^,^29^, suggesting that 53BP1 has a function in the cytoplasm. However, although the nuclear functions of 53BP1 in the DDR and in the choice of DNA repair pathway are well established, the role of 53BP1 in the cytosol remains unknown. In the present study, we used 53BP1 knockdown U2OS, HeLa, knockout H1299 p53-deficient cells, and 53BP1^−/−^ mouse embryonic fibroblasts (MEF) to investigate the physiological importance of 53BP1 in mitochondrial homeostasis, and we provide evidence that 53BP1 regulates the autophagic clearance of damaged mitochondria by promoting parkin translocation to the mitochondria.

## Results

### Loss of 53BP1 leads to mitochondrial aggregation and an increased mitochondrial membrane potential (ΔΨm)

**Figure 1:**
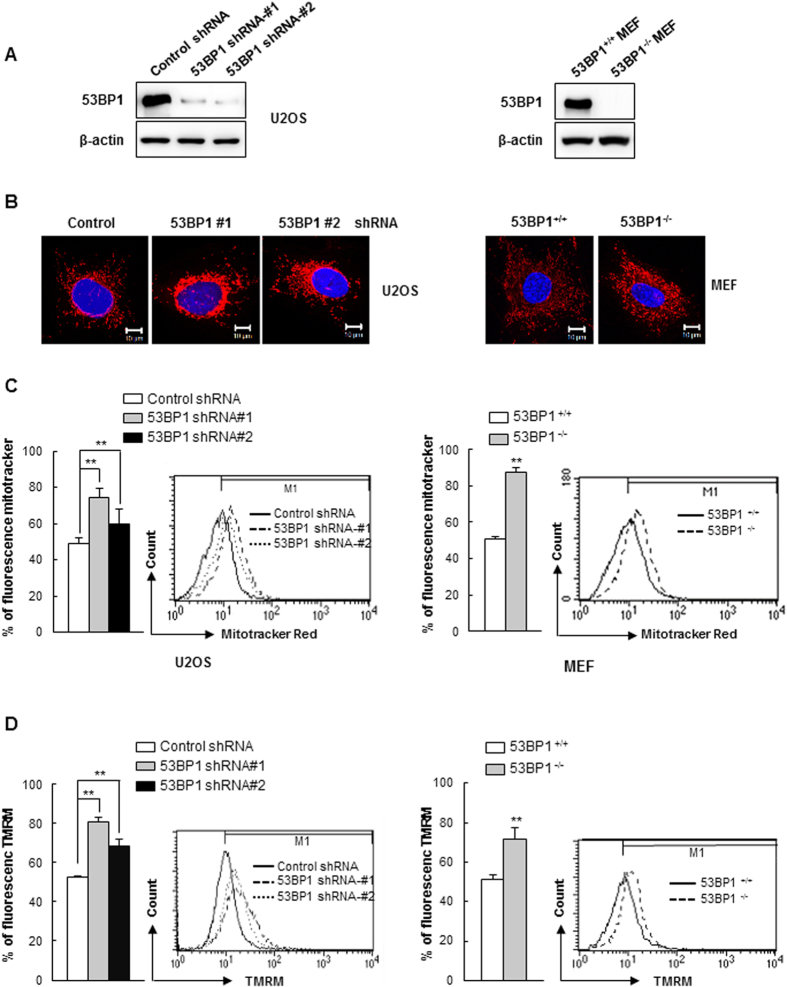
Loss of 53BP1 induces mitochondrial aggregation and increases mitochondrial mass. (**A**) Western blot analysis of 53BP1 from U2OS and MEF cells. Wild type (53BP1^+/+^) and 53BP1 knockout (53BP1^−/−^) MEF cells were prepared from embryos. U2OS cells were stably transfected with either non-targeted shRNA or two different 53BP1-targeted shRNAs. β-actin was included as an internal control. (**B**) Representative cells were stained with MitoTracker Red CMXRos to visualize mitochondria, and fluorescence images were obtained using confocal microscopy. Nuclei were visualized by DAPI staining. (**C**) Cells were incubated with MitoTracker Red CMXRos and the fluorescence was analyzed using flow cytometry. The fluorescence intensity was quantified and the percent change in mitochondrial mass was determined relative to control cells. Histograms from a representative replicate experiment are shown in the right panels. Results are shown as mean ± SD (n = 3), **P < 0.01. (**D**) Cells were incubated with TMRM (Tetramethyl Rhodamine Methyl Ester) for 15 min and intracellular fluorescence intensity was measured using flow cytometry. Fluorescence intensity was then quantified and the percent change in ΔΨm was determined. Results are shown as mean ± SD (n = 3), **P < 0.01.

To investigate the function of 53BP1, we stably depleted U2OS cells using two different shRNAs specific for 53BP1 (Fig. 1A) and visualized the mitochondria of these cells using a mitochondrial-specific, non-cytotoxic dye that accumulates in mitochondria in a membrane potential-dependent manner (Mitotracker Red CMXRos). In control shRNA-transfected cells, staining of the mitochondria was homogenous and appeared to be evenly distributed within the cell, indicative of well-preserved and actively respiring mitochondria (Fig. 1B, left panel). In striking contrast, 53BP1-knockdown cells exhibited an increased intensity of mitochondrial staining and the mitochondria were aggregated. One possible explanation for the differing staining patterns of mitochondria is that the overall mitochondrial content might be different in 53BP1-depleted cells. To test this possibility, we measured the overall mitochondrial mass in both the control and 53BP1-knockdown cells using flow cytometry and found that the 53BP1-depleted cells did indeed have an increased mitochondrial mass (Fig. 1C, left panel). Similar effects were observed when the experiments were repeated using both mouse embryonic fibroblasts (MEFs) from 53BP1 KO mice (Fig. 1A–C, right panels).

The abnormal increase in mitochondrial mass observed in 53BP1-deficient cells could be due to increased mitochondrial biogenesis, decreased clearance of abnormal mitochondria through mitophagy, or both. Several genes involved in mitochondrial biogenesis^30^ were evaluated, but none were expressed at significantly higher levels in 53BP1-deficient cells as compared to control cells (Supplementary Fig. S1), suggesting that mitochondrial biogenesis is not affected. To determine whether or not the rate of mitophagy was affected, we measured mitochondrial membrane potential (ΔΨm), which drops significantly early in the process of mitophagy^10^,^31^. Given that the retention of Mitotracker Red CMXRos by mitochondria depends upon membrane potential, we suspected that a certain subpopulation of mitochondria in 53BP1-depleted cells may have a detectable mitochondrial membrane potential (ΔΨm). To measure the level of ΔΨm depolarization, we stained cells with tetramethyl rhodamine methyl ester (TMRM), which is a cell-permeable fluorescent dye that accumulates in mitochondria in a manner directly proportional to the ΔΨm^32^. We observed that for the 53BP1-knockdown U2OS cells and the 53BP1^−/−^ MEFs, there was an increase in the number of cells with high membrane potentials relative to the controls (Fig. 1D). Mitochondria are selectively engulfed by autophagosomes following a loss in ΔΨm^33^, suggesting that the mitochondrial membrane potential mediates this process. The marked difference in ΔΨm between 53BP1-deficient cells and control cells suggests that the lack of 53BP1 prevented the decline in ΔΨm, thereby preventing mitophagy and resulting in an accumulation of damaged mitochondria.

### 53BP1 regulates mitochondrial function in a p53-independent manner

**Figure 2:**
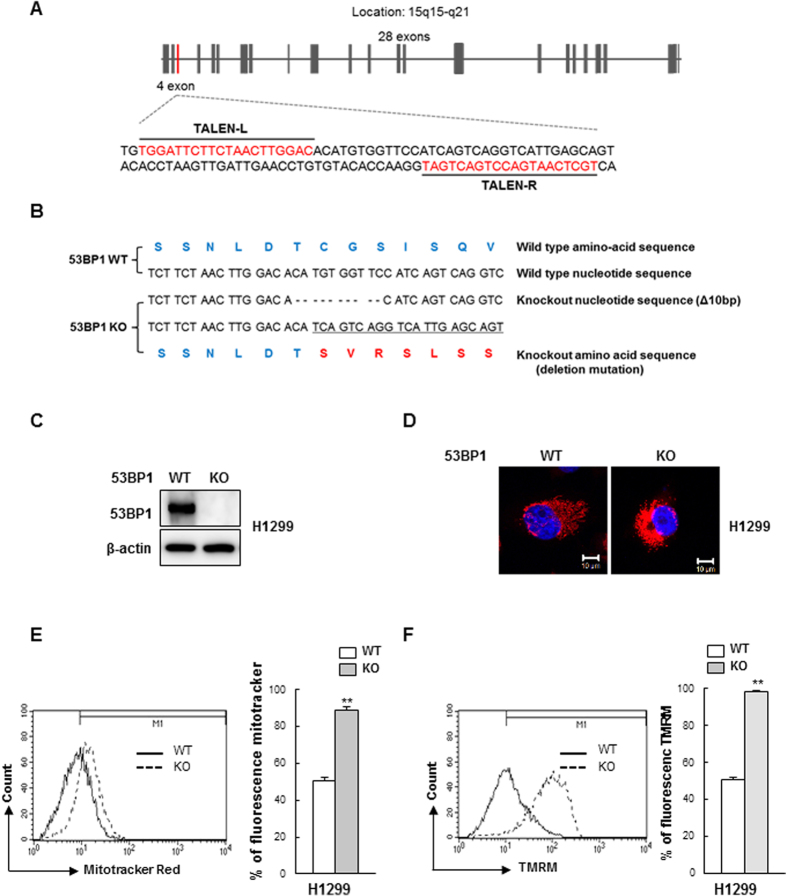
p53 is not required for 53BP1-mediated mitochondrial function. (**A**) A diagram of the strategy for knocking out 53BP1 using the transcription activator-like effector (TALE) endonuclease. Exon 4 of 53BP1 was targeted in the region shown, and the two TALE nuclease binding sequences are underlined. (**B**) Sequencing of the *53BP1* gene from the selected *53BP1* knockout H1299 clone. Dashes indicate deleted bases. The predicted amino acid sequence is indicated based on the recommended description (see Materials and Methods). (**C**) Immunoblot analysis of wild-type cells (WT) and 53BP1 deletion mutant clone (KO). β-actin expression was used as an internal control. (**D**) 53BP1 WT and KO H1299 clones were stained with MitoTracker RedCMXRos and fluorescence images were obtained using confocal microscopy. Nuclei were visualized by DAPI staining. (**E**) 53BP1 WT and KO H1299 clones were incubated with MitoTracker Red CMXRos and mitochondrial mass determination was performed as described in Fig. 1C. Results are shown as mean ± SD (n = 3), **P < 0.01. (**F**) 53BP1 WT and KO H1299 clones were stained with TMRM for 15 min to determine ΔΨm (Mitochondrial membrane potential) as described in Fig. 1D. Results are shown as mean ± SD (n = 3), **P < 0.01.

The tumor suppressor protein p53 plays an important role in mitochondrial homeostasis and inhibits mitophagy^34^,^35^. Because 53BP1 binds to p53 and enhances p53-mediated transcriptional activity, 53BP1-mediated regulation of mitochondrial function might be required for p53 to function properly. To test this hypothesis, we generated 53BP1 knockout (KO) cell lines in p53-deficient non-small-cell lung carcinoma H1299 cells. To generate the 53BP1 KO cell line, we utilized site-directed genetic disruption with engineered TALE nucleases (TALENs)^36^ that targeted exon 4 of the 53BP1 gene (Fig. 2A). Sequencing analysis demonstrated that the loss of 53BP1 expression was due to 10 bp deletion occurring at the genome locus of 53BP1, all of which led to frame shifts and consequently the gene knockout (Fig. 2B). Intact, wild type 53BP1 protein was completely absent in this clone, as measured by Western blotting (Fig. 2C). We then evaluated mitochondrial aggregation in the 53BP1 KO by staining with Mitotracker Red and observing staining intensity via fluorescence microscopy. As expected, there was a greater intensity of MitoTracker Red staining in the 53BP1 KO cells, suggesting that mitochondria were aggregating (Fig. 2D). We followed up by measuring both mitochondrial mass (Fig. 2E) and ΔΨm (Fig. 2F) and observed that both were significantly higher in the 53BP1 KO cells. Similar effects were also observed when the experiments were repeated using the p53-inactivated cervical cancer HeLa cell (Supplementary Fig. S2A,B and C). Taken together, these experiments demonstrate that the role of 53BP1 in mitochondrial function appears to be independent of p53.

### 53BP1 depletion abrogates mitochondrial clearance by autophagy

**Figure 3:**
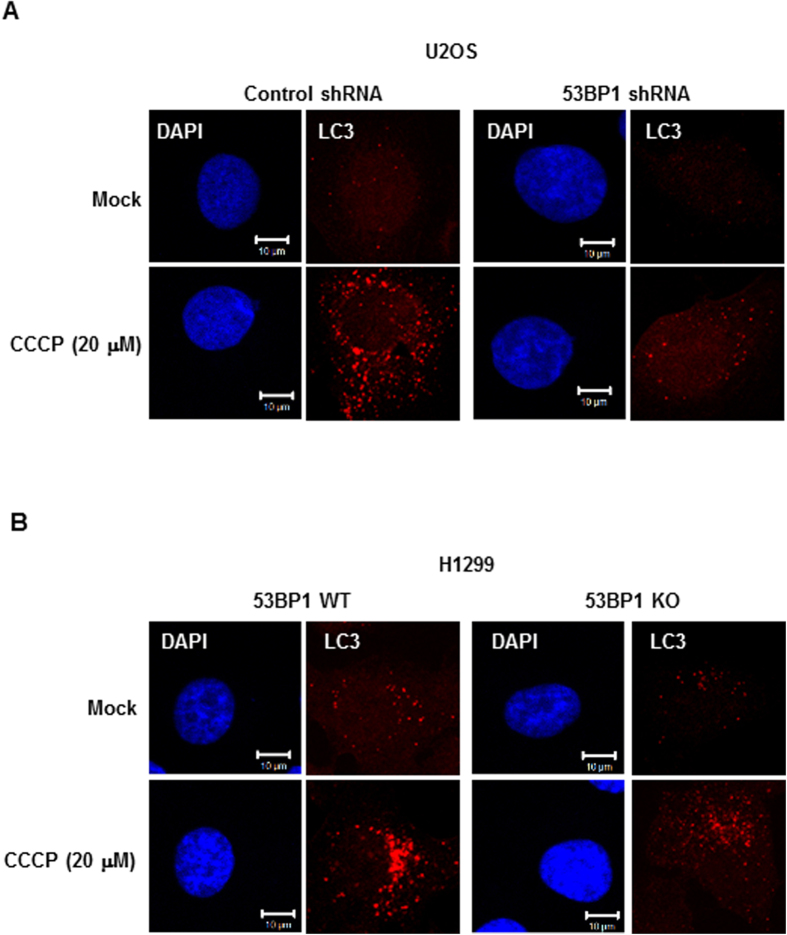
53BP1 plays a role in mitochondrial clearance. Control and 53BP1-deficient U2OS cells (**A**) and 53BP1 WT and KO H1299 cells (**B**) were treated with 20 μM CCCP (Carconyl Cyanide *m*-Chlorophenylhydrazone) for 8 h, and immunostained using an anti-LC3 antibody (red) for autophagosomes and DAPI (blue) for nuclei staining. Images were collected using confocal microscopy.

Selective mitochondrial autophagy, or mitophagy, is the central process by which mitochondrial quality and quantity are maintained^4^. To determine whether or not 53BP1 plays a role in the clearance of damaged mitochondria, we induced mitochondrial damage by treating cells with the mitochondrial uncoupler carbonyl cyanide *m*-chlorophenylhydrazone (CCCP), which is widely used to study mitophagy^37^,^38^, and we assessed the effects by staining for the presence of the autophagosome marker protein LC3. As expected, CCCP treatment of U2OS and HeLa cells resulted in an increase in LC3 intensity as compared to DMSO-treated controls (Fig. 3A and Supplementary Fig. S3A). And notably, there was only a marginal increase in CCCP-induced LC3 staining intensity in 53BP1-knockdown cells. When the experiment was carried out in 53BP1 knockout cells, there was, again, a marked decrease in the number of CCCP-treated autophagosomes as compared to 53BP1 wild-type cells (Fig. 3B and Supplementary Fig. S3B). Thus, defective mitophagy in 53BP1-deficient cells appears to lead to an increase in the number of abnormal mitochondria.

### Removal of damaged mitochondria is impaired in 53BP1-deficient cells

**Figure 4:**
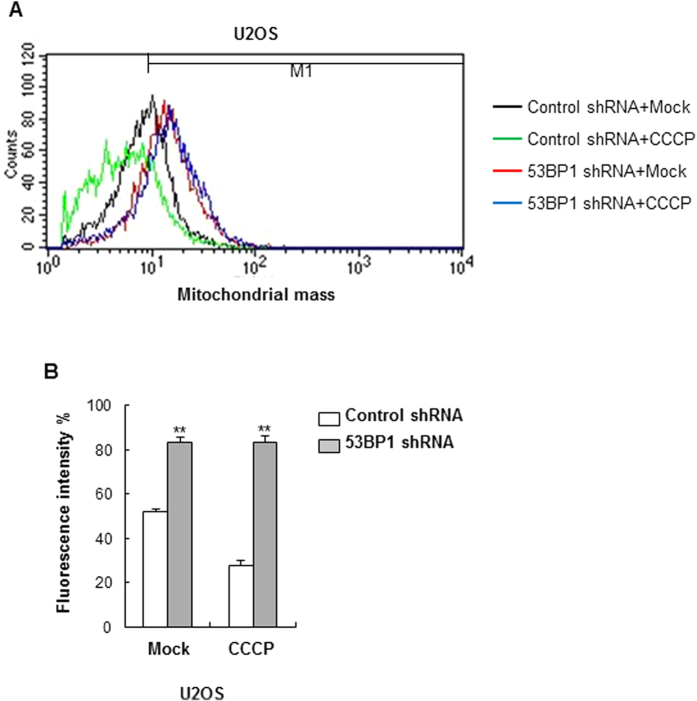
The elimination of damaged mitochondria is impaired in 53BP1-deficient cells. (**A**) Control and 53BP1-deficient U2OS cells were treated with 20 μM CCCP for 24 h and stained with MitoTracker Red CMXRos followed by mitochondrial mass analysis by flow cytometry. Representative histograms of mitochondrial fluorescence intensity are shown. (**B**) Fluorescence intensity of MitoTracker was quantified, and the percent change in mitochondrial mass was determined relative to the control. Results are shown as mean ± SD (n = 3), **P < 0.01.

To look more closely at the role of 53BP1 in mitochondrial clearance via mitophagy, we performed flow cytometry on cells that had been stained with Mitotracker Red CMXRos in the presence or absence of CCCP. As we showed earlier, 53BP1 knockdown increased mitochondrial mass in U2OS cells. The basal mitochondrial mass in control and 53BP1-deficient U2OS cells was 52.2% (±1.05%) and 83.4% (±3.5%), respectively (Fig. 4A,B). Importantly, the mitochondrial mass in U2OS control cells decreased from 52.2% (±1.05%) to 27.9% (±2.07%) after treatment with CCCP. In contrast, there was a high level of mitochondrial mass in both untreated and treated 53BP1-deficient cells, 83.4% (±3.5%) and 83.2% (±2.9%) respectively, indicating that the clearance of abnormal mitochondria via mitophagy is impaired.

### 53BP1 recruits parkin to damaged mitochondria in response to CCCP treatment

**Figure 5:**
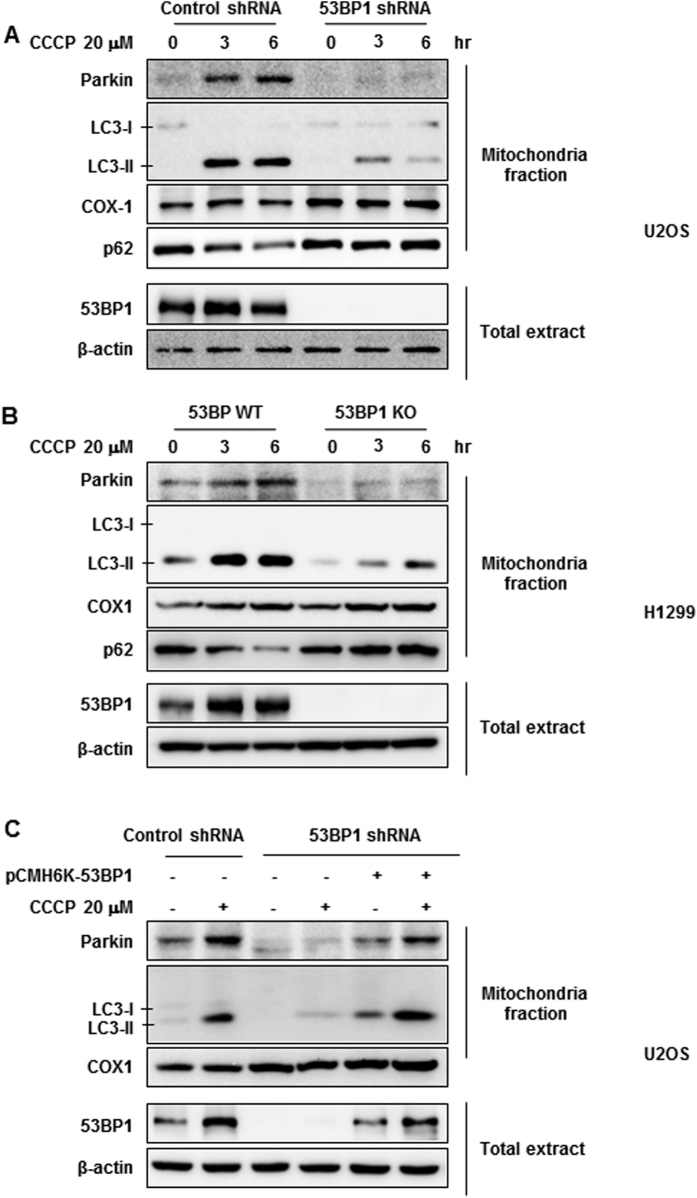
53BP1 affects parkin-mediated mitophagy. Control and 53BP1-deficient U2OS cells (**A**) and 53BP1 WT and 53BP1 KO H1299 cells (**B**) were treated with CCCP for the indicated time points. Total cell extracts and mitochondrial fractions were analyzed by Western blotting with antibodies against 53BP1, Parkin, LC3-II, p62, COX-1, and β-actin as the control. (**C**) 53BP1 stably knockdown U2OS cells were transiently transfected with either the HA-tagged 53BP1 shRNA resistant vector or a control vector (HA-Control). Twenty-four hours after transfection, cells were treated with CCCP for 6 h. Total cell extracts and mitochondrial fractions were then analyzed using the indicated antibodies.

There is evidence that parkin, an E3-ubiquitin ligase, is recruited to damaged mitochondria^37^,^39^, and the induction of mitophagy is dependent on the accumulation of parkin in mitochondria. To determine whether or not the loss of 53BP1 impairs the behavior of parkin, we compared the proteins presented in mitochondrial extracts from 53BP1-knockdown U2OS cells and 53BP1 KO H1299 cells using Western blotting. The presence of COX-1, a mitochondrial specific protein, confirmed the specificity of the mitochondria fraction, and the presence of LC3-II only after CCCP treatment confirmed that CCCP-induced mitochondrial damage results in autophagy (Fig. 5A). CCCP treatment also led to increased amounts of parkin in the mitochondrial fraction (Fig. 5B as had been previously described^37^,^39^. However, the CCCP-induced LC3-II accumulation was impaired in both 53BP1 knockdown U2OS cells and 53BP1-KO H1299 cells (Fig. 5A,B). Furthermore, treatment with CDDP resulted in a downregulation in the level of the p62 protein, which represents a known target for autophagic degradation, however, CDDP-induced downregulation of p62 was significantly suppressed by depletion of a 53BP1 gene, supporting that 53BP1 loss suppresses the autophagic clearance of damaged mitochondria. More interestingly, the lack of 53BP1 significantly suppressed CCCP-induced recruitment of parkin to mitochondria, further supporting the involvement of 53BP1 in the mitophagy.

To further explore the role of 53BP1 in CCCP-induced mitophagy, we reconstituted the 53BP1 knockdown U2OS cells using shRNA-resistant 53BP1 expression vector and followed the effects of 53BP1 reconstitution on the recruitment of parkin to mitochondria. First, we confirmed by immunoblotting that transfection of the shRNA-resistant 53BP1 expression vector did indeed rescue 53BP1 levels (Fig. 5C). We then analyzed mitochondrial levels of both parkin and LC3-II with and without CCCP treatment. As shown in Fig. 5C, after CCCP treatment, LC3-II levels were effectively restored when 53BP1 was present. Of note, the attenuated recruitment of parkin to the mitochondria was almost completely rescued upon the reintroduction of 53BP1 as well, reinforcing this important role for 53BP1 in parkin-mediated mitophagy.

## Discussion

53BP1 protein is predominantly a nuclear protein that plays an important role in the DDR signaling pathway and in DNA repair^24^,^25^,^26^,^27^. It has been also reported that 53BP1 is present in, or even restricted to, the cytoplasm of both mouse embryo fibroblasts and COS-1 cells^28^,^29^. However, the role of the small fraction of 53BP1 that exists outside of the nucleus remains largely undefined. In the present study, we provide the first evidence that 53BP1 plays an important role in the autophagy-dependent clearance of dysfunctional mitochondria from the cytoplasm.

To directly address how mitochondrial function is affected in 53BP1-depleted cells, we first compared the structural organization of mitochondria in control versus 53BP1 shRNA-transfected U2OS cells, as well as wild-type versus 53BP1^−/−^ MEF cells, using the mitochondrial-specific stain Mitotracker Red CMXRos and we found marked differences. Both strains lacking 53BP1 had darkly stained mitochondria that tended to be aggregated. We suspected that the difference in staining between the control and 53BP1-deficient cells may have resulted from an inability of the mutant strain to clear abnormal mitochondria. Indeed, 53BP1-deficient cells contained a significantly larger mass of mitochondria. Changes in mitochondrial membrane potential are an early marker of mitophagy^4^, and when we compared the mitochondrial membrane potential between control and 53BP1-deficient cells using the fluorescence dye TMRM, we found that 53BP1-knockdown U2OS cells and 53BP1^−/−^ MEF cells both had a higher potential than control cells, again suggesting that the loss of 53BP1 may hinder mitophagy.

It is known that the p53 protein not only regulates apoptosis but also autophagy. There is evidence that p53 promotes mitochondrial dysfunction by inhibiting Parkin-mediated mitophagy^34^,^35^. Because 53BP1 was identified as a p53 binding protein and enhances p53-mediated transcriptional activation^40^,^41^, it was possible that the relationship between 53BP1 deficiency and mitochondrial dysfunction was associated with the status of p53. However, in the present study, a disruption in 53BP1 expression in p53-deficient H1299 cells and p53-inactivated HeLa cells still resulted in an induction of mitochondrial aggregation, increased mitochondrial mass, and increased mitochondrial membrane potential, indicating that the effect of 53BP1 depletion on the number of dysfunctional mitochondria was independent of p53.

Given the putative role of 53BP1 in mitophagy, a more detailed analysis of autophagosomes was necessary. Using the uncoupler CCCP to induce damage to mitochondria, we demonstrated that LC3, a protein that binds to mitochondria early in mitophagy, fails to accumulate in mitochondria to wild type levels in a 53BP1 knockdown-U2OS cells and a 53BP1 knockout-H1299 cells. Typically the parkin protein translocates to depolarized mitochondrial membranes and induces mitophagy thereby maintaining mitochondrial homeostasis^37^,^39^. Our study showed that CCCP-induced translocation of parkin to mitochondria was inhibited by the depletion of 53BP1 in U2OS and H1299 cells. Furthermore, we show that the translocation of parkin to mitochondria is independent of p53. Consistently, overexpression of 53BP1 in the deficient strains restored both LC3 accumulation and parkin translocation to mitochondria. Together these findings support the hypothesis that 53BP1 plays an important role in the the autophagic clearance of damaged mitochondria by promoting parkin translocation to the mitochondria.

Although this is the first report demonstrating that 53BP1 contributes to mitophagy, several questions remain regarding how mitophagy is actually regulated by the 53BP1. 53BP1 regulates DNA double-strand breaks repair through its association with several proteins^42^. Thus, it is possibility that cytosolic 53BP1 could bind directly to one of the mitophagy-related proteins, resulting in the regulation of removal damaged mitochondria. It has also been reported that 53BP1 enhance p53-mediated transcriptional activation^40^,^41^. However, in this study, using p53-deficient H1299 cells and p53-inactivated HeLa cells, we showed that the role of 53BP1 in mitophagy is independent of p53. Therefore, 53BP1 may regulate mitophagy-related gene expression in a p53-independent manner. Accordingly, studies aimed at determining the detailed mechanisms underlying 53BP1 regulation of mitophagy are currently under way.

## Methods

### Cell lines

Wild-type (B6; 129J) and 53BP1-knockout mice (B6; 129-Trp53bp1^tmlJC^/J) were purchased from JAX^®^ Mice (The Jackson Laboratory; Bar Harbor, ME, USA). Mouse embryonic fibroblasts (MEFs) were prepared from embryonic day 13.5 embryos and maintained in Dulbecco’s modified Eagle medium (DMEM, Invitrogen, Carlsbad, CA, USA) supplemented with 10% fetal bovine serum (FBS, Invitrogen), 100 units/ml of penicillin, 100 μg/ml of streptomycin (100 × Pen/Strep solution, Invitrogen) and nonessential amino acids (100 × NEAA solution, Invitrogen). U2OS cells were cultured in McCoys 5A (Invitrogen) containing 10% FBS and penicillin-streptomycin. HeLa cells were maintained in DMEM with 10% FBS and penicillin-streptomycin. P53-deficient human non-small lung carcinoma cell line H1299 cells were cultured in RPMI-1640 with 10% FBS and penicillin-streptomycin. All cells were cultured in a humidified incubator with 5% CO2 at 37 °C. All cell lines were from the American Type Culture Collection (ATCC) and were grown according to standard protocols.

### Ethics statement

The experimental protocol was approved by the Institution Animal Care and Use Committee (IACUC) of Chosun University School of Medicine (IACUC No. CHOSUN- 2015-12-001) and complied with the NIH *Guide for “The Care and Use of Laboratory Animals*”. The animals were housed in specific pathogen-free (SPF) rooms with free access to food and water.

### shRNA vector and 53BP1 knockdown cell line

10 × 10^5^ U2OS cells were seeded in 60mm dishes 24 hours before transfection. The cells were transiently transfected 53BP1 siRNA or control siRNA using Lipofectamine RNAiMax (Invitrogen) according to the manufacturer’s instructions. The sequences of the 53BP1 siRNAs were: GCC AGG TTC TAG AGG ATG A (53BP1 siRNA-#1) and GGA CAA GTC TCT CAG CTA T (53BP1 siRNA-#2). These siRNAs and negative control siRNA were purchased from Bioneer (Daejeon, Korea). The reduction of 53BP1 protein expression was analyzed by Western blotting. To generate stable 53BP1-depleted cell lines, oligonucleotides were designed and synthesized as follows: 53BP1 top: 5′-GAT CCG GAC AAG TCT CTC AGC TAT TTC AAG AGA ATA GCT GAG AGA CTT GTC CTT TTT TGG AAA-3′; bottom: 5′-AGC TTT TCC AAA AAA GGA CAA GTC TCT CAG CTA TTC TCT TGA AAT AGC TGA GAG ACT TGT CCG-3′. The oligonucleotides were annealed and ligated into the linearized pSilencer 2.1-U6 neo shRNA expression vector (Ambion^®^ Thermo Fisher Scientific, Waltham, MA, USA). 2 × 10^5^ U2OS and 2.5 × 10^5^ HeLa cells were seeded in 35mm dishes 24 hours before transfection. The cells were transfected with pSilencer-53BP1 shRNA expression vector or control shRNA expression vector using Lipofectamine 2000 (Invitrogen). All vectors contained the neomycin resistance gene. After 48 h, 0.5 mg/ml neomycin was added to the culture medium for selection of the transfected cells, and these culture conditions were used for 2–3 weeks. Cell lines that had stably silenced 53BP1 expression were confirmed by Western blot analysis.

### Preparation of TALEN-mediated 53BP1 knockout cells

TALEN editing used to generate 53BP1 knockout cells. The TALEN-Human H27702 vector kit for 53BP1 targeting was purchased from ToolGen (Seoul, Korea) and then 53BP1 knockout cells were prepared according to manufacturer’s instructions. The 53BP1 target sequence is 5′-TGG ATT CTT CTA ACT TGG ACA CAT GTG GTT CCA TCA GTCAGG TCA TTG AGC A-3′ as described in Fig. 2A. The vector kit contains Human H27702-TALEN-L vector: expression plasmid with TALEN pair left specific for the target site, 5′-TGG ATT CTT CTA ACT TGG AC-3′, Human H27702-TALEN-R vector: expression plasmid with TALEN pair right for the target site, 5′-TGC TCA ATG ACC TGA CTG AT-3′ and GFP-Reporter vector. Briefly, 2 × 10^5^ H1299 cells were seeded in 35 mm dishes 24 hours before transfection. The cells were co-transfected with TALEN-L, TALEN-R and reporter vector using Lipofectamine 2000 (Invitrogen). 48 h after transfection, the twenty cells were seeded and cultured during 2–3 weeks, and then colonies were selected. In order to assess 53BP1 expression, genomic DNA was isolated to perform DNA sequencing, or protein extracts were lysed to perform Western blotting. TALEN-mediated mutations were confirmed by sequencing. Clones lacking expression of 53BP1 were used for further experiments.

### 53BP1 shRNA resistant 53BP1 expression vector

The pCMH6K 53BP1 expression vector was obtained from Kuniyoshi Iwabuchi (Kanazawa Medical University, Japan). To construct the shRNA-resistant 53BP1 expression vector, we performed mutagenesis using GeneArt Site-Directed Mutagenesis Kits (Invitrogen) in accordance with the manufacturer’s instructions. We designed sequences for shRNA resistance: sense: 5′-CGC ACA TCA AGT GGG ACT AGC CTG TCT GCT ATG CAC AGC AGT-3′ and antisense: 5′-ACT GCT GTG CAT AGC AGA CAG GCT AGT CCC ACT TGA TGT GCG-3′. Site-directed mutations of the 53BP1 shRNA-resistant HA-53BP1 expression vector were confirmed by sequencing.

### Mitochondrial morphology

The indicated cells (3 × 10^5^ cells/well) were seeded in 6-well plate 24 hours before stain. The cells were stained with 100 nM MitoTracker Red CMXRos (Invitrogen) for 15 min at 37 °C on coverslips, then fixed with 4% paraformaldehyde in phosphate-buffered saline (PBS) for 15 min and washed three times in PBS. The cells were mounted on glass slides by using fluorescent Mounting Medium with DAPI (GBI Labs, Mukilteo, WA, USA). Images were acquired using a Zeiss LSM 510 Meta confocal microscope (Carl Zeiss, Weimar, Germany) with a 63 × 1.4 oil objective analyzed with the Zeiss microscope image software ZEN (Carl Zeiss).

### Flow cytometry analysis for mitochondria

3 × 10^5^ cells were seeded in 6-well plate 24 hours before staining. Mitochondrial mass was measured by fluorescence levels upon staining with 100 nM MitoTracker Red CMXRos (Invitrogen) for 15 min at 37 °C. Mitochondrial membrane potential (ΔΨm) was measured by staining cells with 100 nM TMRM (Invitrogen) for 15 min at 37 °C. Cells were then washed with PBS solution, trypsinized and re-suspended in PBS. Mitochondrial mass was analyzed by flow cytometry (FACSCalibur, BD Biosciences, San Jose, CA, USA) using the FL2 detector. Log scale fluorescence histograms were analyzed for median relative fluorescent unit (RFU) intensity using CellQuest Pro software. The data are depicted as the mean ± s.d. value in independent experiments.

### Immunofluorescence confocal microscopy

Cells (3 × 10^5^ cells/6-well plate) were cultured on cover slips coated with poly-L-lysine (Sigma-Aldrich, St. Louis, MO, USA) and were treated with or without CCCP for 8 h. Cells were washed in PBS, and fixed in methanol for 5 min at room temperature, then incubated blocking buffer (1% BSA in PBS). Incubation of rabbit polyclonal anti-LC3B antibody (1:500, Cell Signaling, Danvers, MA, USA) was performed overnight at 4 °C. The cells were washed three times in PBS and incubated with secondary antibody (Alexa Fluor 594 chicken anti-rabbit, 1:200, Invitrogen) in blocking buffer for 2 h. Cell were washed three times in PBS and mounted in Fluorescent Mounting Medium with DAPI (GBI Labs). Images were acquired on a Zeiss LSM 510 Meta confocal microscope (Carl Zeiss) and analyzed with Zeiss microscope image software ZEN (Carl Zeiss).

### Protein extraction

For total protein extraction, cells (2–3 × 10^6^) were lysed in ice-cold NP-40 buffer [50 mM Tris-HCl (pH 7.5), 150 mM NaCl, and 1% Nonidet P-40, 0.5% sodium deoxycholate, 0.1% sodium dodecyl sulfate, 1 mM dithiothreitol, 1 mM phenylmethanesulfonyl fluoride, 10 μg/ml leupeptin and 10 μg/ml aprotinin] on ice for 10 min. For extraction of the mitochondrial fraction, 8–9 × 10^6^ cells were suspended in cytosol buffer [20 mM Hepes, 10 mM KCl, 1.5 mM MgCl_2_, 1 mM EDTA, 1 mM EGTA, 1 mM DTT, 1 mM phenyl methane sulfonyl fluoride, 10 μg/ml leupeptin, 10 μg/ml pepstatin A and 10 μg/ml aprotinin], and were lysed by passing them through a 25-gauge needle 20 times. The lysate was centrifuged at 13,000 rpm for 10 min at 4 °C to remove cell debris and nuclei. The supernatants were centrifuged at 80,000 rpm for 15 min at 4 °C. The pellet, corresponding to the mitochondrial fraction, was lysed in ice cold NP-40 lysis buffer [50 mM Tris-HCl (pH 7.5), 150 mM NaCl, and 1% Nonidet P-40, 0.5% sodium deoxycholate, 0.1% sodium dodecyl sulfate, 1 mM dithiothreitol, 1 mM phenylmethanesulfonyl fluoride, 10 μg/ml leupeptin and 10 μg/ml aprotinin] on ice for 10 min. The mitochondria fractions were sonicated twice for 2 s, and then centrifuged at 13,000 × g for 20 min. The protein amount was determined using the Bradford protein assay (Bio-Rad, Hercules, CA, USA).

### Western blotting

Each fraction was measured for protein concentration using a Bradford protein assay (Bio-Rad, Hercules, CA, USA), and 20 μg of proteins was separated by 6–12% SDS-PAGE, and transferred to a polyvinylidene difluoride membrane (PALL life sciences, Washington, NY, USA). The membrane was blocked for 1 h with blocking solution [5% skim milk in TBS-T (10 mM Tris-HCl (pH 7.4), 150 mM NaCl, 0.1% Tween-20] and then incubated with primary antibodies overnight at 4 °C. The following primary antibodies were used: rabbit polyclonal anti-53BP1 (1:1000, Santa Cruz, Dallas, TX, USA), mouse monoclonal anti-Parkin (1:1000, Santa Cruz), mouse monoclonal anti-β-actin (1:2000, Santa Cruz), goat polyclonal anti-COX-1 (1:1000, Santa Cruz), mouse monoclonal anti-LC3 (1:1000, NanoTools, Munchen, germany), purified mouse anti-p62 lck ligand (1:1000, BD Biosciences, San Jose, CA, USA). The membranes were washed three times in TBS-T buffer and then incubated for 2 hours with horseradish peroxidase (HRP)-conjugated secondary antibodies (1:4000, Jackson Laboratory, West Grove, PA, USA). After rinsing three times, the membrane was treated with ECL detection reagents (WEST-ZOL plus, iNtRON Biotechnology, Korea) and the specific bands were visualized on a luminescent image analyzer LAS-4000mini (Fujifilm Life Science, Stamford, CT, USA).

### Statistical analysis

Data in all experiments are presented as mean ± standard deviation (SD) of three independent results. Statistical comparisons were carried out using two-tailed paired Student *t*-test. We considered *P* < 0.01 (indicated as ** in Figures) as significant.

## Additional Information

**How to cite this article**: Youn, C. K. *et al*. 53BP1 contributes to regulation of autophagic clearance of mitochondria. *Sci. Rep.* **7**, 45290; doi: 10.1038/srep45290 (2017).

**Publisher's note:** Springer Nature remains neutral with regard to jurisdictional claims in published maps and institutional affiliations.

## Supplementary Material

Supplementary Information

## References

[b1] Westermann B. Mitochondrial fusion and fission in cell life and death. Nat Rev Mol Cell Biol 11, 872–884, doi: 10.1038/nrm3013 (2010). 21102612

[b2] Wallace D. C., Fan W. & Procaccio V. Mitochondrial energetics and therapeutics. Annu Rev Pathol 5, 297–348, doi: 10.1146/annurev.pathol.4.110807.092314 (2010). 20078222 PMC3245719

[b3] Chan D. C. Mitochondria: dynamic organelles in disease, aging, and development. Cell 125, 1241–1252, doi: 10.1016/j.cell.2006.06.010 (2006). 16814712

[b4] Youle R. J. & Narendra D. P. Mechanisms of mitophagy. Nat Rev Mol Cell Biol 12, 9–14, doi: 10.1038/nrm3028 (2011). 21179058 PMC4780047

[b5] Pieczenik S. R. & Neustadt J. Mitochondrial dysfunction and molecular pathways of disease. Exp Mol Pathol 83, 84–92, doi: 10.1016/j.yexmp.2006.09.008 (2007). 17239370

[b6] Petersen K. F. . Mitochondrial dysfunction in the elderly: possible role in insulin resistance. Science 300, 1140–1142, doi: 10.1126/science.1082889 (2003). 12750520 PMC3004429

[b7] Exner N., Lutz A. K., Haass C. & Winklhofer K. F. Mitochondrial dysfunction in Parkinson’s disease: molecular mechanisms and pathophysiological consequences. Embo j 31, 3038–3062, doi: 10.1038/emboj.2012.170 (2012). 22735187 PMC3400019

[b8] Wu J. J. . Mitochondrial dysfunction and oxidative stress mediate the physiological impairment induced by the disruption of autophagy. Aging (Albany NY) 1, 425–437 (2009). 20157526 10.18632/aging.100038 PMC2806022

[b9] Kim I. & Lemasters J. J. Mitophagy selectively degrades individual damaged mitochondria after photoirradiation. Antioxid Redox Signal 14, 1919–1928, doi: 10.1089/ars.2010.3768 (2011). 21126216 PMC3078512

[b10] Ashrafi G. & Schwarz T. L. The pathways of mitophagy for quality control and clearance of mitochondria. Cell Death Differ 20, 31–42, doi: 10.1038/cdd.2012.81 (2013). 22743996 PMC3524633

[b11] Jacobs H. T. & Turnbull D. M. Nuclear genes and mitochondrial translation: a new class of genetic disease. Trends Genet 21, 312–314, doi: 10.1016/j.tig.2005.04.003 (2005). 15922826

[b12] Shimizu I., Yoshida Y., Suda M. & Minamino T. DNA damage response and metabolic disease. Cell Metab 20, 967–977, doi: 10.1016/j.cmet.2014.10.008 (2014). 25456739

[b13] Correia-Melo C. . Mitochondria are required for pro-ageing features of the senescent phenotype. EMBO J 35, 724–742, doi: 10.15252/embj.201592862 (2016). 26848154 PMC4818766

[b14] Akbari M., Sykora P. & Bohr V. A. Slow mitochondrial repair of 5′-AMP renders mtDNA susceptible to damage in APTX deficient cells. Sci Rep 5, 12876, doi: 10.1038/srep12876 (2015). 26256098 PMC4530458

[b15] Brand M. D. The sites and topology of mitochondrial superoxide production. Exp Gerontol 45, 466–472, doi: 10.1016/j.exger.2010.01.003 (2010). 20064600 PMC2879443

[b16] Liu P. & Demple B. DNA repair in mammalian mitochondria: Much more than we thought? Environ Mol Mutagen 51, 417–426, doi: 10.1002/em.20576 (2010). 20544882

[b17] Yakes F. M. & Van Houten B. Mitochondrial DNA damage is more extensive and persists longer than nuclear DNA damage in human cells following oxidative stress. Proc Natl Acad Sci USA 94, 514–519 (1997). 9012815 10.1073/pnas.94.2.514 PMC19544

[b18] Alexeyev M. F., Ledoux S. P. & Wilson G. L. Mitochondrial DNA and aging. Clin Sci (Lond) 107, 355–364, doi: 10.1042/CS20040148 (2004). 15279618

[b19] Greaves L. C., Reeve A. K., Taylor R. W. & Turnbull D. M. Mitochondrial DNA and disease. J Pathol 226, 274–286, doi: 10.1002/path.3028 (2012). 21989606

[b20] Szczepanowska J., Malinska D., Wieckowski M. R. & Duszynski J. Effect of mtDNA point mutations on cellular bioenergetics. Biochim Biophys Acta 1817, 1740–1746, doi: 10.1016/j.bbabio.2012.02.028 (2012). 22406627

[b21] Schultz L. B., Chehab N. H., Malikzay A. & Halazonetis T. D. p53 binding protein 1 (53BP1) is an early participant in the cellular response to DNA double-strand breaks. J Cell Biol 151, 1381–1390 (2000). 11134068 10.1083/jcb.151.7.1381 PMC2150674

[b22] Celeste A. . Histone H2AX phosphorylation is dispensable for the initial recognition of DNA breaks. Nat Cell Biol 5, 675–679 (2003). 12792649 10.1038/ncb1004

[b23] Fernandez-Capetillo O. . DNA damage-induced G2-M checkpoint activation by histone H2AX and 53BP1. Nat Cell Biol 4, 993–997, doi: 10.1038/ncb884 (2002). 12447390

[b24] Chapman J. R., Sossick A. J., Boulton S. J. & Jackson S. P. BRCA1-associated exclusion of 53BP1 from DNA damage sites underlies temporal control of DNA repair. J Cell Sci 125, 3529–3534, doi: 10.1242/jcs.105353 (2012). 22553214 PMC3445322

[b25] Bunting S. F. . 53BP1 inhibits homologous recombination in Brca1-deficient cells by blocking resection of DNA breaks. Cell 141, 243–254, doi: 10.1016/j.cell.2010.03.012 (2010). 20362325 PMC2857570

[b26] Escribano-Diaz C. . A cell cycle-dependent regulatory circuit composed of 53BP1-RIF1 and BRCA1-CtIP controls DNA repair pathway choice. Mol Cell 49, 872–883, doi: 10.1016/j.molcel.2013.01.001 (2013). 23333306

[b27] Panier S. & Boulton S. J. Double-strand break repair: 53BP1 comes into focus. Nat Rev Mol Cell Biol 15, 7–18, doi: 10.1038/nrm3719 (2014). 24326623

[b28] Gonzalez-Suarez I. . A new pathway that regulates 53BP1 stability implicates cathepsin L and vitamin D in DNA repair. EMBO J 30, 3383–3396, doi: 10.1038/emboj.2011.225 (2011). 21750527 PMC3160650

[b29] Iwabuchi K. . Stimulation of p53-mediated transcriptional activation by the p53-binding proteins, 53BP1 and 53BP2. J Biol Chem 273, 26061–26068 (1998). 9748285 10.1074/jbc.273.40.26061

[b30] Valentin-Vega Y. A. . Mitochondrial dysfunction in ataxia-telangiectasia. Blood 119, 1490–1500, doi: 10.1182/blood-2011-08-373639 (2012). 22144182 PMC3286212

[b31] Ly J. D., Grubb D. R. & Lawen A. The mitochondrial membrane potential (deltapsi(m)) in apoptosis; an update. Apoptosis 8, 115–128 (2003). 12766472 10.1023/a:1022945107762

[b32] Jobe S. M. . Critical role for the mitochondrial permeability transition pore and cyclophilin D in platelet activation and thrombosis. Blood 111, 1257–1265, doi: 10.1182/blood-2007-05-092684 (2008). 17989312 PMC2214770

[b33] Lemasters J. J. . The mitochondrial permeability transition in cell death: a common mechanism in necrosis, apoptosis and autophagy. Biochim Biophys Acta 1366, 177–196 (1998). 9714796 10.1016/s0005-2728(98)00112-1

[b34] Hoshino A. . Cytosolic p53 inhibits Parkin-mediated mitophagy and promotes mitochondrial dysfunction in the mouse heart. Nat Commun 4, 2308, doi: 10.1038/ncomms3308 (2013). 23917356

[b35] Hoshino A. . Inhibition of p53 preserves Parkin-mediated mitophagy and pancreatic beta-cell function in diabetes. Proc Natl Acad Sci USA 111, 3116–3121, doi: 10.1073/pnas.1318951111 (2014). 24516131 PMC3939874

[b36] Gaj T., Gersbach C. A. & Barbas C. F. 3rd. ZFN, TALEN, and CRISPR/Cas-based methods for genome engineering. Trends Biotechnol 31, 397–405, doi: 10.1016/j.tibtech.2013.04.004 (2013). 23664777 PMC3694601

[b37] Narendra D., Tanaka A., Suen D. F. & Youle R. J. Parkin is recruited selectively to impaired mitochondria and promotes their autophagy. J Cell Biol 183, 795–803, doi: 10.1083/jcb.200809125 (2008). 19029340 PMC2592826

[b38] Suen D. F., Narendra D. P., Tanaka A., Manfredi G. & Youle R. J. Parkin overexpression selects against a deleterious mtDNA mutation in heteroplasmic cybrid cells. Proc Natl Acad Sci USA 107, 11835–11840, doi: 10.1073/pnas.0914569107 (2010). 20547844 PMC2900690

[b39] Vives-Bauza C. . PINK1-dependent recruitment of Parkin to mitochondria in mitophagy. Proc Natl Acad Sci USA 107, 378–383, doi: 10.1073/pnas.0911187107 (2010). 19966284 PMC2806779

[b40] Derbyshire D. J. . Crystal structure of human 53BP1 BRCT domains bound to p53 tumour suppressor. Embo j 21, 3863–3872, doi: 10.1093/emboj/cdf383 (2002). 12110597 PMC126127

[b41] Joo W. S. . Structure of the 53BP1 BRCT region bound to p53 and its comparison to the Brca1 BRCT structure. Genes Dev 16, 583–593, doi: 10.1101/gad.959202 (2002). 11877378 PMC155350

[b42] Callen E. . 53BP1 mediates productive and mutagenic DNA repair through distinct phosphoprotein interactions. Cell 153, 1266–1280, doi: 10.1016/j.cell.2013.05.023 (2013). 23727112 PMC3713552

