# Supplementary material for: 53BP1 contributes to regulation of autophagic clearance of mitochondria
Source: Sci Rep. 2017 Mar 27;7:45290. doi: 10.1038/srep45290 (PMC5366885; doi:10.1038/srep45290)

Supplementary Information

53BP1 contributes to regulation of autophagic clearance of mitochondria

Cha Kyung Youn [1,2], Hong Beum Kim[1,2], Ting Ting Wu[1,5], Sang-Gon Park[3,*], Sung Il Cho [4,*], and Jung-Hee Lee[1,2,*]

[1]Laboratory of Genomic Instability and Cancer therapeutics, Cancer Mutation Research Center, [2]Department of premedical Sciences, [3]Department of Internal Medicine, Hemato-oncology, [4]Department of Otolaryngology-Head and Neck Surgery, [5]Department of Cellular and Molecular Medicine, Chosun University School of Medicine, 375 Seosuk-dong, Gwangju 61452, Republic of Korea.

Running Title: 53BP1 regulates mitophagy

Key Words: 53BP1, Parkin, mitophagy, mitochondrial homeostasis

Conflict of interest: The authors have declared that no conflict of interest exists.

Cha Kyung Youn and Hong Beum Kim contributed equally to this work.

*Address Correspondence To:
Jung-Hee Lee, Ph.D.
Phone, 82-62-230-6399; Fax, 82-62-230-6586; Email: jhlee75@chosun.ac.kr

Sung Il Cho, M.D., Ph.D.
Phone, 82-62-220-3207; Fax, 82-62-225-2702; E-mail: chosi @chosun.ac.kr

Sang-Gon Park, M.D., Ph.D.
Phone, 82-62-220-3984; Fax, 82-62-227-9653; E-mail: sgpark@chosun.ac.kr

Supplementary Figure legends

Figure S1. 53BP1 knockdown is not associated with increased mitochondrial biogenesis. Expression of genes involved in mitochondrial biogenesis is intact in control and 53BP1-deficient U2OS cells. PGC1β, TFAM, PPARα, HPRT, CYCS, and ERRα mRNAs were measured by real-time qPCR analyses. mRNA levels were normalized using GAPDH mRNA as an internal control. Results are shown as mean ± SD (n = 3). n.s. indicated not statistically significant.

Figure S2. 53BP1 knockdown leads to mitochondrial aggregation and increases mitochondrial mass in HeLa cells. (A) Western blot analysis of 53BP1 from control and 53BP1-deficient HeLa cells. HeLa cells were stably transfected with either non-targeted shRNA or 53BP1-targeted shRNA. β-actin was included as an internal control. (B) Representative cells were stained with MitoTracker Red CMXRos to visualize mitochondria, and fluorescence images were obtained using confocal microscopy. Nuclei were visualized by DAPI staining. (C) Cells were incubated with MitoTracker Red CMXRos and the fluorescence was analyzed using flow cytometry. The fluorescence intensity was quantified and the percent change in mitochondrial mass was determined relative to control cells. Histograms from a representative replicate experiment are shown in the lower panels. Results are shown as mean ± SD (n = 3), $^{**}P<0.01$. (D) Cells were incubated with TMRM for 15 min and intracellular fluorescence intensity was measured using flow cytometry. Fluorescence intensity was then quantified and the percent change in $\Delta\Psi m$ was determined. Results are shown as mean ± SD (n = 3), $^{**}P<0.01$.

Figure S3. 53BP1 plays a role in mitochondrial clearance. Control and 53BP1-deficient HeLa cells (A) and wild-type ($53BP1^{+/+}$) and 53BP1 KO ($53BP1^{-/-}$) MEFs (B) were treated with CCCP for 8 h, and immunostained using an anti-LC3 antibody (red) for autophagosomes and DAPI (blue) for nuclei staining. Images were collected using confocal microscopy.

Supplementary methods

Quantitative real time PCR (RT-qPCR)

Total RNA from 10×10^5 U2OS cells was isolated using the Trizol reagent (Invitrogen) and reverse transcribed into cDNA using Reverse Transcriptase M-MLV (Takara, Mountain View, CA, USA) according to the manufacturer's protocol. Real-time PCR analysis was performed using the SYBR green-based fluorescent method (SYBR premix Ex Taq kit, TaKaRa) and the MX3000P® qRT-PCR system (Stratagene, La Jolla, CA, USA) with specific primers. Primers used for real-time PCR are as follows: *PGC1β* forward, 5'- GCC TCT CCA GGC AGG TTC A-3' and *PGC1β* reverse, 5'-TAG AGA ACT CAG TCC AGA AGG CTT T-3'; *TFAM* forward, 5'- CGG CAG AGA CGG TTA AAA AAG-3' and *TFAM* reverse, 5'- TTC CCT GAG CCG AAT CAT CC-3'; *PPARα* forward, 5'- GCG TAC GGC AAT GGC TTT AT-3' and *PPARα* reverse, 5'-GAA CGG CTT CCT CAG GTT CTT-3'; *HPRT* forward, 5'- GCT CGA GAT GTG ATG AAG GAG AT-3' and *HPRT* reverse, 5'-AGC AGG TCA GCA AAG AAT TTA TAG C-3'; *CYCS* forward, 5'- GCA AGC ATA AGA CTG GAC CAA A -3' and *CYCS* reverse, 5'-TTG TTG GCA TCT GTG TAA GAG AAT C-3'; *ERRα* forward, 5'-GCA GGG CAG TGG GAA GCT A-3' and *ERRα* reverse, 5'-CCT CTT GAA GAA GGC TTT GCA-3', *GAPDH* forward, 5'- TTC ACC ACC ATG GAG AAG GC-3' and *GAPDH* reverse, 5'-GGC ATG GAC TGT GGT CAT GA-3'. The quantity of transcripts was calculated based on the threshold cycle (C_t) using the delta-delta C_t method

that measures the relative concentration of a target RNA between two samples by comparing them to the level of a normalization RNA control, *GAPDH*.

Supplementary Figure S1

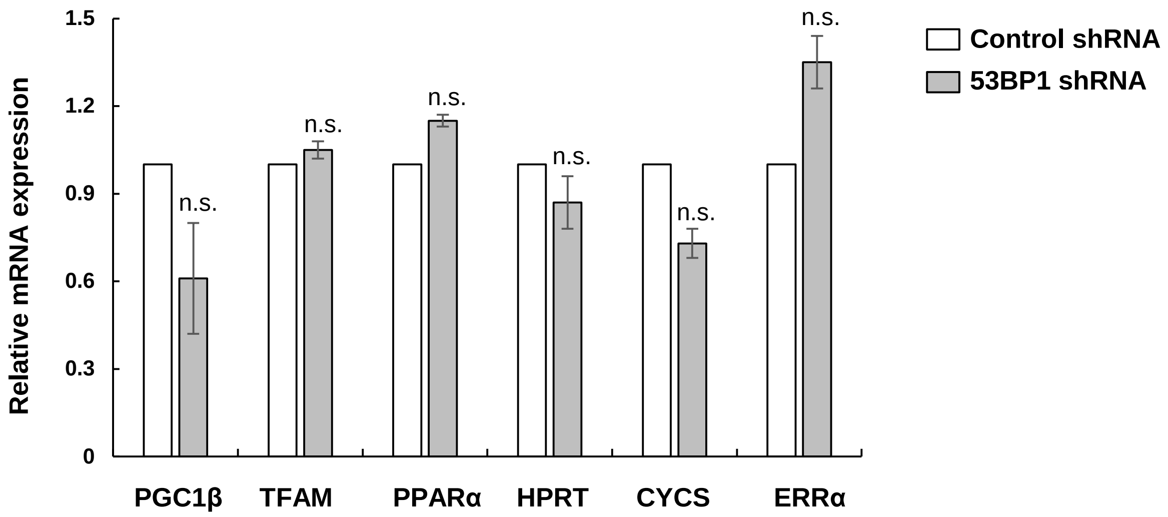

Supplementary Figure S2

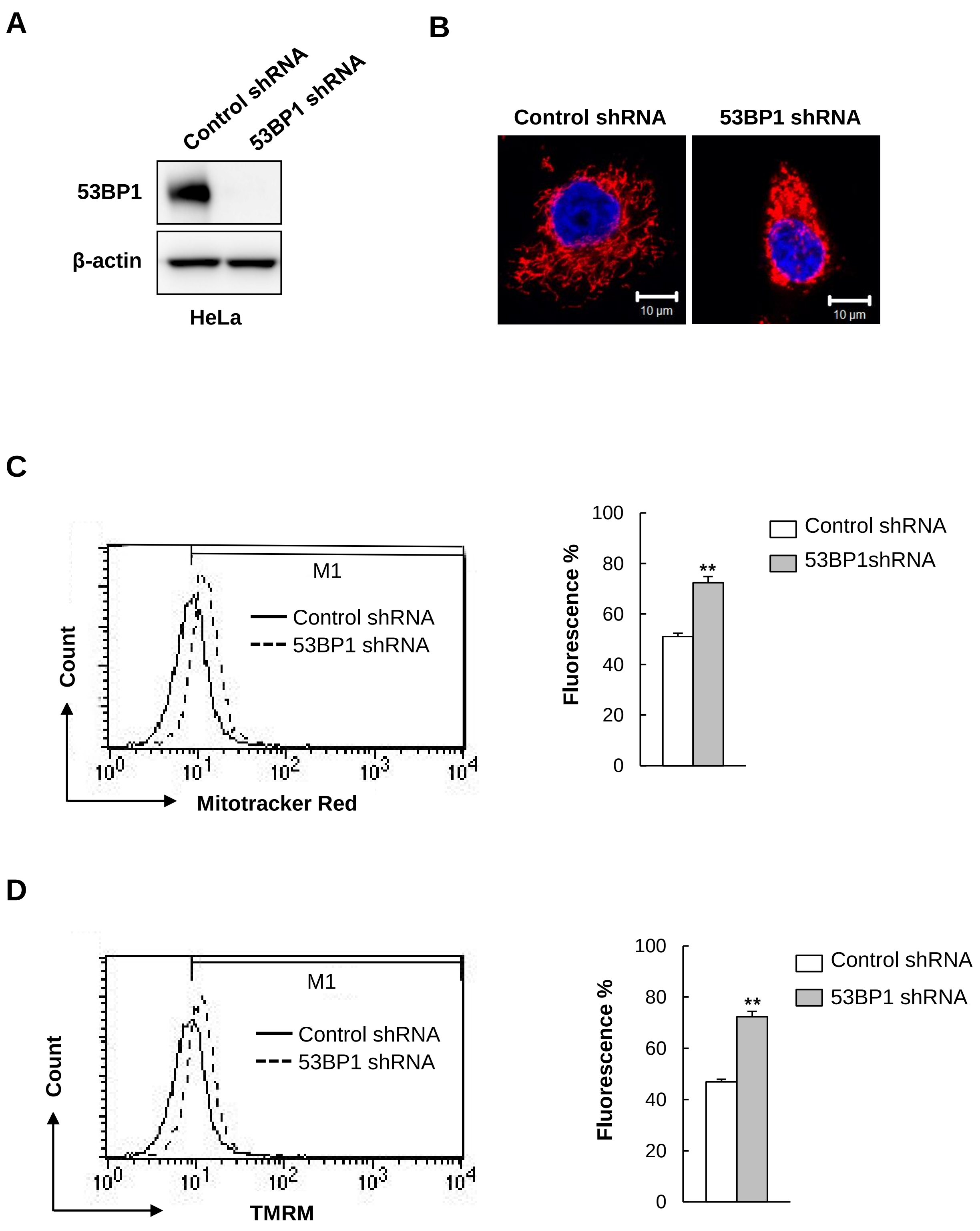

Supplementary Figure S3

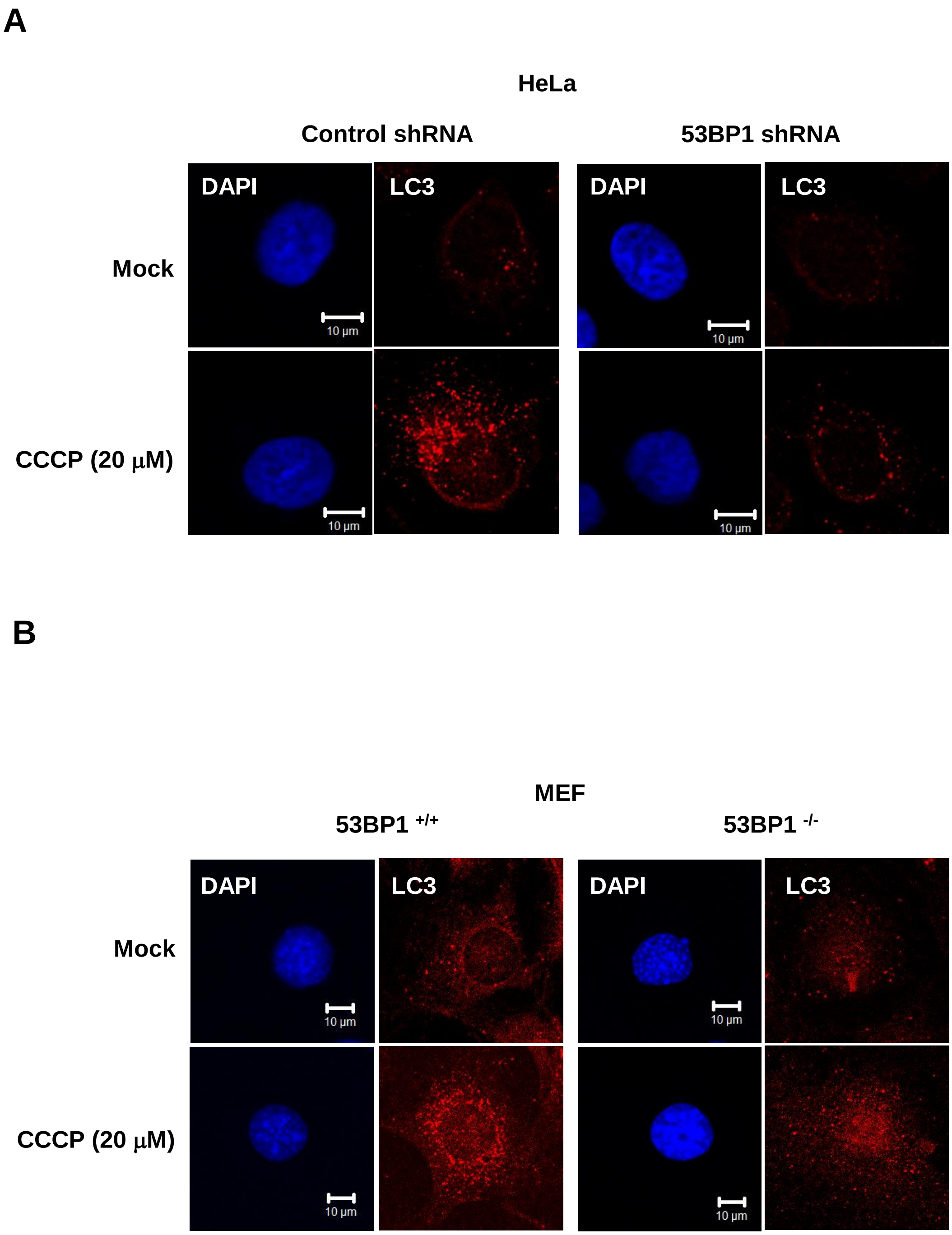

Supplement: Supplementary Information [file srep45290-s1.pdf]
